# Sulphate of Quinia in the Congestive Modifications of Scarlet Fever and Measles

**Published:** 1846-08

**Authors:** Daniel Stahl

**Affiliations:** Quincy, Illinois


ILLINOIS AND INDIANA

MEDICAL AND SURGICAL JOURNAL.

Vol. I. NEW SERIES.—AUGUST, 1846. No. 3.

### PART I.—ORIGINAL COMMUNICATIONS.

### ARTICLE I.

*Sulphate of Quinia in the Congestive Modifications of Scarlet Fever and Measles.* By Daniel Stahl, M. D., of Quincy, Illinois.

It has long been questioned, whether medicine, in the true sense of the word, is a *Science* or not, and however many there may be, that claim for it a place in the circle of Sciences, it is to my mind doubtful whether it deserves it. As long as we have not incontrovertible data to go upon; as long as we cannot with mathematical certainty explain the phenomena of life; as long, I say, as we do not proceed from *positive principles,* so long can it not claim a place in the company of Sciences. It is, at least, entitled to the appellation of an *Experimental Science.* It draws from experience, and refers scattered facts and phenomena, to general laws. But as long as it has to deduce, or discover these hidden laws from appreciable phenomena, so long is it encumbered with hypotheses, which we all know are very unsafe until the mass of phenomena, satisfactorily explained thereby, become so numerous that the explanation has ceased to be an hypothesis. While, however, we thus support one hypothesis after another, and while by the multiplicity of observations and experiments, we discover, that that, which was but yesterday an hypothesis, to be to-day considered a law of nature, we at the same time discover new laws, and throw new light upon a department of nature the most hidden in its operations, and the most beneficial in its practical applications. Whoever, therefore, multiplies the mass of isolated facts, whoever communicates his experience and observations, especially if new, contributes to the improvement of medicine and becomes a benefactor to his race. Every Physician of many years practice will admit, that the character of diseases changes, that remedies which he in former years administered with success, are now either inert or injurious, and that therefore, he has to look about for a new remedy, or apply an old remedy in a new disease. If he has the good fortune to hit upon a remedy, which answers the purpose, he ought to let it be known, in order to afford those who have the talents, and opportunities, means to answer the whys and wherefores. The same is applicable to discoveries of new remedies. These remarks may probably subject me to the reproach, that I put our profession too low in respect to its claims as a science; be it so: I will not defend myself, but only ask those who thus reproach me; "Can you in every case, when you administer a dose of calomel, say, what are the pathological changes you wish to remove, and what is, in this case, the modus operandi of the calomel? Do you never administer calomel or any other remedy for no other reason, but because it had a salutary effect in a similar case? And do you not call this, in your inmost thoughts empiricism?"

In medicine we want no idle words, nor talk for talking's sake; we want facts, true observations, and laws and principles deduced from them. The Homœpathist will prove by the handsomest sophistry in the world, that a grain of Balladonna diluted and shaken 20 times up and down, is enough to destroy a whole community, yet we all know that experience proves the contrary. Dr. Franklin, in an answer to the enquiry of a wag, laid down a principle, that we as medical men ought to pursue. A wag, in Paris I think, asked him the reason why a piece of solid iron, made in the shape of a fish, would swim in water; the sagacious Doctor answered him, "Let me see a solid piece of iron thus shaped, swim, and afterwards I will endeavor to find an explanation." Let therefore every new fact, every new observation be recorded and give thereby those who have the talents, materials for a systematic arrangement.

These preliminary remarks I have intended not only as an apology for troubling my readers with the account of the effects of so well known a medicinal agent as Quinine, but also as an argument to the members of our profession to prove, that we, as private practitioners, can benefit mankind as much, if not more, by communicating isolated facts, as by searching constantly for theories and hypotheses.

*Sulphate of Quinia* has been looked upon as a *tonic*, and until within a very recent date, no practitioner would venture to administer it in any case during febrile excitement, or when sordes in the primæ viæ were suspected to be present, except in *cases of great emergency.* But these few cases of emergency, in which it has been given *successfully*, teach a lesson that should not be lost sight of. They have shown us, that Quinine is not a mere tonic, in the common acceptation of the term, that it does not excite the vascular system, but that it is a *nervine* in the strictest sense of the word, that it merely gives tone and energy to the nerves if prostrated, and that it thereby assuages the undue excitement, consequent, not upon an hyperdynamic, but more upon an adynamic condition of the system, and consisting of, an effort of the weakened powers, to regulate the vital functions, which for this purpose require more energy of the nerves, than they in such cases possess. Such a condition it is, which I consider, constitutes what we improperly call a *Congestive State* or *Congestive Fever;* and such is the condition, with which the practitioner in this country has often to battle, in autumnal bilious fever, pneumonia notha, (vulgarly called winter fever,) scarlet fever, and measles. Formerly, physicians were led astray in the treatment of congestive fever, and judging from the term "congestion," mistook the pathology of this modification of diseases, particularly of bilious fever, and in accordance with the advise of Armstrong, bled profusely, and frequently, no doubt, bled their patients to death. Happily has the introduction of inductive reasoning into our practice convinced us of the error of our predecessors, in this respect; we have found that depletion cannot remove this condition which is called congestion, but that a remedy which has been designated as a tonic (I mean the Quinine,) not only effects this, but also, and at the same time, instead of accelerating, diminishes the frequency of the pulse.

I never have seen many cases of the congestive modification of *Scarlet fever,* such as are described in books, particularly such as Eberle describes; but in the epidemic which prevailed here last fall and last winter, I had occasion to see many cases attended with "oppression, deep seated pain and a sense of weight in the head, fullness and great muscular prostration," with "quick, short respiration." In these cases the pulse was not "slow, irregular and weak," but quick, soft and regular. In such cases I gave the Quinine in large and often repeated doses, with the happiest effects. If the skin was pale or livid, I gave the Quinine with capsicum; but as the most of these cases in our last epidemic exhibited only oppression, prostration, quick pulse, and red and warm skin, I gave the quinine alone.

I have obtained the most remarkable and prompt effect from Quinine, in the *congestive modification of measles,* the most prominent features of which are *unusually quick, short* and *anxious respiration,* and *quick pulse.* I will relate the history of three cases of this kind, which I have attended, and as I do it principally from memory, I hope for the reader's indulgence for the imperfections of the pictures I shall present.

Case I. Mary Carter, aged 12, a healthy girl of rather a nervous temperament, was taken on the 10th of March, 1846, with the initiatory symptoms of measles, such as coryza, inflamed conjunctiva, cough, &c., for which I ordered my usual prescription in such cases, an emetic of Ipecacuanha. The next day the measles made their appearance in the usual way, and with the usual symptoms. In the night, from the 13th to the 14th, the parents discovered that she drank more than common, and had great difficulty of breathing. In the morning of March 14th, I was called, and found her, as far as I can recollect, with the following symptoms: measles over the whole body, red eyes, *respiration very quick, short and laborious,* so that she had to be propped in bed, and complained constantly of want of air; the chest was heaving, the nostrils dilated, and the alæ nasi playing, and the whole appearance presented the picture of a dreadful struggle with death. Patient withal was perfectly conscious of this, and spoke, with great difficulty, of the imminent danger in which she was placed. Much thirst; *pulse* 140 *in a minute,* regular, soft, but not very weak, nor small; constipation; skin warm. There was great oppression in the scrobiculus cordis, so that deep inspiration was impossible.

I prescribed a large dose of calomel and gr. ii of quinine, with ½ gr. of opium every ¾ to ½1 hours. With every dose of these powders, the breathing improved and pulse became slower.

*March* 15*th*. Bowels moved by means of injectives. Breathing easier, and pulse 120 in a minute; can lay lower with her head. Continued with the above powders every 1½ hour, and, as she became better, every 2 hours.

*March* 16*th*. Drinks less, slept some, pulse slower. Continued the powders every 2 to 3 hours. Under this treatment in conjunction with the prescription of some adjuvants, such as mustard draughts, blisters to the chest, warm applications and cathartics, my patient entirely recovered.

Case II. *March* 3*d*. Fred. Beck, aged 4, a strong, healthy, German boy, who had never been seriously ill, except an attack of scarlet fever, was taken in the usual way with the measles, which in the usual time made their appearance on the skin, and extended from the face downwards, over the whole body. In the night, from the 2d to the 3d of March, his parents discovered some difficulty of breathing. In the morning I was called and found that the eruption maintained the usual (normal) color, *but the breathing was so short and quick*, and *withal so laborious*, that it alarmed me. All the muscles contributing to the act of respiration were in full play, and the lower jaw was at each inspiration, drawn down. The pulse was rather soft, small, and so quick that I could hardly count it. I gave him immediately *quinine in grain doses* every hour, and in 24 hours from the time I was first called, I considered him out of danger.

Case III. *March* 31*st*. T. Blanke, 7 years of age, who has always enjoyed excellent health, of a stout, robust frame, was taken in the usual way with the measles, which in due course of time made their appearance. On the 31st of March I was called; I found his skin very hot, exanthema partly disappeared, severe hard cough, *breathing short*, *quick and laborious*, *pulse very quick and full*, but not hard, thirst excessive, constipation. Under any other circumstances, and had I not seen the above two cases, I should have depleted this boy, but fearing venesection, I ordered a cathartic, and a solution of tart. antim. in nauseating doses. The next day the fever had diminished, but the pulse was, if anything, quicker than before, the breathing worse, and the cough not much better. I now commenced the use of quinine, by which he improved so much that after he had taken it about 20 hours, I gave him some of Coxe's Hive Syrup, and soon dismissed him cured.

